# An overlapping module identification method in protein-protein interaction networks

**DOI:** 10.1186/1471-2105-13-S7-S4

**Published:** 2012-05-08

**Authors:** Xuesong Wang, Lijing Li, Yuhu Cheng

**Affiliations:** 1 School of Information and Electrical Engineering, China University of Mining and Technology, Xuzhou, Jiangsu 221116, P. R. China

## Abstract

**Background:**

Previous studies have shown modular structures in PPI (protein-protein interaction) networks. More recently, many genome and metagenome investigations have focused on identifying modules in PPI networks. However, most of the existing methods are insufficient when applied to networks with overlapping modular structures. In our study, we describe a novel overlapping module identification method (OMIM) to address this problem.

**Results:**

Our method is an agglomerative clustering method merging modules according to their contributions to modularity. Nodes that have positive effects on more than two modules are defined as overlapping parts. As well, we designed de-noising steps based on a clustering coefficient and hub finding steps based on nodal weight.

**Conclusions:**

The low computational complexity and few control parameters prove that our method is suitable for large scale PPI network analysis. First, we verified OMIM on a small artificial word association network which was able to provide us with a comprehensive evaluation. Then experiments on real PPI networks from the MIPS Saccharomyces Cerevisiae dataset were carried out. The results show that OMIM outperforms several other popular methods in identifying high quality modular structures.

## Background

In general, a good understanding of protein families provides us with further views on biological processes. Previous studies have shown that modular structures are densely connected internally but sparsely interacting with others in PPI networks [1,2]. Modules can be understood as independent sub-networks and proteins in the same module always interact more frequently and show stronger functional dependencies. These days, more and more people are likely to address biological problems with graphic models, where proteins or genes are viewed as nodes and their pair wise interactions as edges in a network [3,4].

Several methods have been proposed for module identification in the last decade. In 2003, Bader and Hogue proposed a molecular complex detection method (MCODE), which can separate densely connected regions by assigning a weight to each protein [5]. A Markov clustering method (MCL) which is based on flow simulation and high-flow areas corresponding to protein complexes was applied to detect protein families in 2002 [6]. A network module mining method (NeMo) proposed by Yan *et al*. identifies frequent dense sub-graphs in input networks using coherent edge frequencies, which can lose statistical power in sparse networks with few edges [7]. However, most of the existing methods cannot identify overlapping modules in PPI networks. As far as we know, some proteins may be included in multiple complexes and component parts of a complex could be activated at a specific time or location [8,9].

In 2006, a clique percolation method (CPM) was used for the first time to identify overlapping modules in PPI networks by finding fully connected sub-graphs of different minimum clique sizes [10]. But its high computational complexity (O(exp(*n*))where *n *represents the number of nodes in the network) hindered its application to large scale networks.

Based on these considerations, we propose the OMIM, which is able to partition large scale PPI networks with overlapping modular structures. OMIM first clusters all nodes using a Newman algorithm [11] and then defines nodes that have comparatively positive effects on the modularity of more than two modules as overlapping ones. Moreover, we designed de-noising steps through assigning a weight to each edge. Hubs can also be found according to their nodal weight. OMIM is a method that is able to identify highly interconnected modules and has few control parameters, allowing it to be applied to many types of networks. We evaluate OMIM as applied to an artificial network and a PPI network. The results showed that it outperforms several other current methodologies.

## Methods

### Overview

As we know, a PPI network can be described as an undirected and unweighted graph, *G*=(*V,E*), where *V *and *E *represent nodes (proteins) and edges (interactions) in the network. In our method, we first assign weights to all edges according to their importance to the network and remove those with lower weights as noise. Then the steps for identifying overlapping modules are performed. The main idea of identifying overlapping parts in OMIM is to find nodes that have comparatively positive effects on different modules. In addition, hubs were also found according to connections with their neighbors [12].

### De-noising

In general, data in PPI networks are obtained from high-throughput protein-protein interaction experiments. So far, the most frequently used protein-protein interaction detection methods are yeast-2-hybrid, tandem affinity purification, mass spectrometry technology and protein chip technology. Although these high-throughput detection methods make for easy experimentation, they bring about noise and incompleteness [13-15].

The main idea in our de-noising step is to assign a weight to each edge of a PPI network to reflect the reliability of the corresponding interactions. In our study, we use a popular metric from graph theory, i.e., clustering coefficient. A clustering coefficient is a measure that represents the interconnectivity in the neighborhood of a node [16]. The clustering coefficient of node *i *with degree *k_i _*can be described as

(1) $$CC_{i}=\frac{2n_{i}}{k_{i}\left(k_{i}-1\right)}$$

where *n_i _*denotes the number of triangles that go through node *i*.

The weight between nodes *i *and *j *can be assigned according to the following equation:

(2) $$SCC\left(i,j\right)=CC_{i}+CC_{j}-CC_{i}^{\prime }-CC_{j}^{\prime }$$

where *CC*' represents the clustering coefficient after the edge between *i *and *j *is removed. According to the viewpoint of Asur et al. [16], if two nodes are not actually connected in the original network, then the *SCC*(*i,j*) value should be small or equal to zero. Here, we define a threshold *α*, and remove edges that are smaller than *α *as noise.

(3) SCC(i,j)≤α

### Overlapping module identification method

#### Newman algorithm

Because OMIM is a variant of the Newman algorithm, we first introduce the Newman algorithm briefly. This is a hierarchical agglomerative method based on the idea of modularity [11]. We know that modularity is a measure of the quality of a particular division of a network and a large value of modularity always corresponds to good network division [17]. If we let *e_rk _*be the fraction of edges in the network, connecting nodes in group *r *to those in group *k *and let $a_{r}=\underset{k}{\sum }e_{rk}$, then

(4) $$Q=\underset{r}{\sum }e_{rr}-a_{r}^{2}$$

where *Q *is a quality function representing modularity. The physical meaning of Eq. (4) is that modularity is equal to the fraction of edges that fall within modules, minus the expected value of the same quantity if edges fall at random without regard to its modular structure [11]. The Newman algorithm is a method for optimizing *Q *in order to discover the best modular structure.

The steps of the Newman algorithm can be summarized as follows.

Step 1. Initialize each node in the input data to be a module, define a matrix *e *and a vector *a *according to Eqs. (5) and (6).

(5) $$e_{ij}=\left\{\begin{array}{cc} 1/2m, & \text{nodes}\;i\;\text{and}\;j\text{ are connected}\\ 0, & \text{else} \end{array}\right)$$

(6) $$a_{i}=k_{i}/2m$$

where *m *represent the total number of edges in the network.

Step 2. Calculate the change of modularity Δ*Q *according to:

(7) $$\Delta Q=2\left(e_{ij}-a_{i}a_{j}\right)$$

Merge module pairs with the maximum value of Δ*Q*. Update matrix *e *by adding the rows and columns of the corresponding merged modules.

Step 3. Repeat Step 2, until the entire network has become one big module.

From this description, the progress of the Newman algorithm can be represented as a dendrogram. If we choose to cut at different levels, different modular structures can be obtained. Actually, Newman chooses to cut at the maximum value of *Q *to obtain the best modular structure.

### Identifying overlapping parts

It should be noted that complexes in PPI networks are not static and proteins can be included in different modules. Therefore, identifying overlapping parts between different modules is necessary. We first perform the Newman algorithm to the input data. Then we try to identify overlapping nodes according to their contribution to modularity. The detailed steps are as follows.

Step 1. Perform Newman algorithm. All nodes are clustered without overlapping parts.

Step 2. Define nodes, whose neighbors belong to more than two modules, to be candidate nodes.

Step 3. Randomly select node *i *from the set of candidate nodes. Assume that *i *is in module *A *and one of its neighbors,*j*, in module *B*. Copy *i *to *B *and a new module *B*' is obtained. If Eq. (8) is satisfied, then *i *is an overlapping node.

(8) $$Q_{B^{\prime }}>Q_{B}$$

where *Q_B _*and *Q_B' _*is the modularity of *B *and *B'*.

Step 4. Repeat Steps 2 ~ 3 until all overlapping parts are identified.

### Discovering hubs

Jordan et al. first found hubs when they studied the evolution of protein and referred to the proteins with large number of partners as hubs [18]. Han et al. divided hubs into two classes: party hubs and date hubs [19]. Party hubs are hubs that interact with their partners at the same time, whereas date hubs either bind their different partners at different times or at different locations. According to their study in a network with a modular structure, date hubs always organize the proteome, while party hubs function inside modules. We propose a computational method to detect the hubs far easier.

First, we defined party hubs as those proteins that have maximal nodal weight (*w_i_*) in a module, i.e.,

(9) $$w_{i}=\underset{j}{\sum }SCC\left(i,j\right),\;j\in \left\{neighbor\;of\;i\right\}$$

(10) $$party\;hub_{r}=\text{arg}\text{max}w_{i}\;i\in r,$$

where *partly hub_r _*means a party hub of module *r*.

Date hubs are defined as proteins that bind at least three modules. We set a variable *ACC_i _*to denote the number of modules to which *i *is bound. The computational method of *ACC_i _*is

(11) $$ACC_{i}=\overset{n_{r}}{\underset{r=1}{\sum }}f\left(i\right)$$

where *n_r _*is the total number of modules in the network and *f*(*i*) is defined as follows:

(12) $$f\left(i\right)=\left\{\begin{array}{c} 1,\;i\;\text{connect}\;\text{to}\;\text{at}\;\text{least}\;\text{one}\;\text{node}\;\text{in}\;r\\ 0,\text{ else} \end{array}\right)$$

### Algorithm

1. de-noising

input: *G*=(*V,E*); *α*

for all nodes *i*(*i*∈*V*) in *G*

compute the clustering coefficient *CC_i_*

end

for all edges (*i,j*)((*i,j*)∈*E*) in *G*

compute the weight *SCC*(*i,j*)

if *SCC*(*i,j*)<*α*

remove edge (*i,j*) as noise

end

end

a new graph *G*'=(*V*',*E'*) is obtained

2. clustering

input: *G*'=(*V*',*E'*); number of nodes *n*; number of edges *m*

compute degree *k *for all nodes and construct *e *and *a*

$$e_{ij}=\left\{\begin{array}{cc} 1/2m, & \text{nodes}\;i\;\text{and}\;j\text{ are connected}\\ 0, & \text{else} \end{array}\right)$$

$$a_{i}=k_{i}/2m$$

1. compute the increment of modularity Δ*Q *for all edges

$$\Delta Q=2\left(e_{ij}-a_{i}a_{j}\right)$$

2. while (there are more than one modules)

merge the module pairs with the maximum Δ*Q*;

update *e *and *a*;

recalculate Δ*Q*;

end

3. sort all *Q *s from all iterations and choose the modular structure *M *corresponding to the largest *Q*.

4. for node *i *in *M*

if *i *belongs to module *A *and its neighbor (in *G*') *j *belongs to *B*

copy *i *to *B *and construct *B'*

if $Q_{B^{\prime }}>Q_{B}$

*i *is an overlapping node between *A *and *B*

end

end

end

5. a new modular structure *M*' with overlapping parts is obtained.

3. discovering hubs

input: *M*'

for module *r *in *M*'

party hub_r_=argmax w_i_,i∈r

end

for each node *i *not in any module

if *ACC_i_*≥3

*i *is a date hub

end

end

## Results and discussion

### Data sources

**Figure 1:**
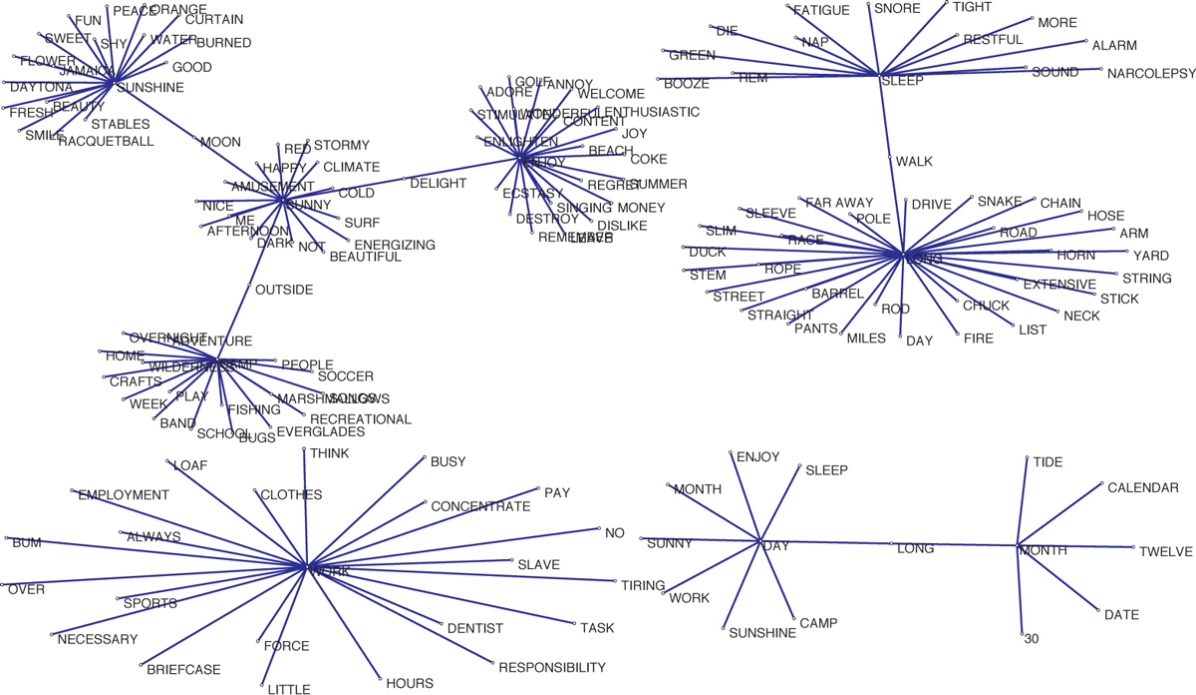
**Artificial word association dataset**. The artificial word association dataset is a small scale network used to validate OMIM. It can be seen as a double layer network. 9 words constitute the first layer, in which the word 'day' works as a hub. The second layer consists of 8 sub-networks that center on other 8 words in the first layer, i.e., month, sunshine, camp, sleep, work, enjoy, long and sunny.

In our experiments, we validated our method on two datasets, i.e., a small-scale artificial dataset and a large-scale PPI dataset. The artificial dataset is derived from the South Florida Word Association database [20], with 151 nodes and 155 edges in the network (Figure 1). The eight core nodes playing important roles are month, sunshine, camp, sleep, work, enjoy, long and sunny respectively, which are connected by the key word day.

The yeast (Saccharomyces Cerevisiae) PPI networks used in our study are from the MIPS Comprehensive Yeast Genome Database (CYGD) (PPI_18052006) [21]. The dataset contains 4989 proteins and 13583 interactions after removing isolated nodes and self-cycled edges. The on-line annotation tool, GO term finder (version 0.83), is from the SGD database (Saccharomyces Genome Database) [22], which contains 7292 genes as a background set.

Methods used for comparisons in our experiments are Newman, MCL and CPM. There are two main reasons for this selection. In first instance, these are three classical clustering algorithms that have been widely used in many fields. Their use makes for clearer comparisons. Secondly, these algorithms represent the most appropriate methods in different aspects for comparison with OMIM. According to Brohée et al. [23], MCL outperforms many other algorithms, especially in partitioning PPI networks. CPM is a widely known classical method for identifying overlapping modules and the Newman algorithm is the ancestor of OMIM.

Among these three methods, MCL was executed as an embedded program of BioLayout Express 3D [24] and the CPM algorithm was performed by using of CFiner, a tool created for clustering based on CPM [25].

### Performance on an artificial dataset

Three evaluation indices, i.e., accuracy (AC), overlapping rate (OL) and average degree (AVD) were used.

(13) $$AC=\overset{n}{\underset{i=1}{\sum }}\overset{m_{i}}{\underset{j=1}{\sum }}x_{i}\left(j\right)/)n$$

(14) $$OL=\overset{n_{r}}{\underset{r=1}{\sum }}num\text{\_}V\left(r\right)/)n$$

(15) $$AVD=2\overset{n_{r}}{\underset{r=1}{\sum }}num\text{\_}E\left(r\right)/)\overset{n_{r}}{\underset{r=1}{\sum }}num\text{\_}V\left(r\right)$$

where node *j *is a neighbor of node *i*, *m_i _*represents the total number of neighbor nodes of *i*, *num_V(r) *and *num_E(r) *represent the number of nodes and edges in module *r *respectively. *x_i_*(*j*) is a function defined as follows: if *j *is classified correctly, *x_i_*(*j*)=1; else, *x_i_*(*j*)=0.

Table 1 shows that the OMIM performed better than the other methods on accuracy. Although CPM is an algorithm which is able to find overlapping modular structures, it performed worst on the artificial dataset. The reason for this is that, the CPM filtered too much useful nodes during its execution. MCL discovered one more module than OMIM. The discrepancy is primarily due to the fact that MCL cannot deal with hierarchical networks and regards the first layer as another module. Note that the OL value of Newman is 1, which is a result of its inability to identify overlapping module structures.

**Table 1:** Results of the comparison on the word association dataset

Algorithm	AC	OL	AVD	NUM_M	D_hub	P_hub
OMIM	1.0000	1.0265	1.9817	8	1	8
Newman	0.9810	1.0000	1.8904	8	-	-
MCL	0.9934	1.0063	2.0132	9	-	-
CPM	0.0043	0.0199	0.0397	1	-	-

AC: accuracy. OL: overlapping rate. AVD: average degree. D_hub: date hub. P_hub: party hub. NUM_M: the number of modules obtained by different methods. '-': a symbol meaning we were unable to discover party or date hubs.

Eight party hubs were found by OMIM, i.e., month, sunshine, camp, sleep, work, enjoy, long and sunny. The date hub is day. Besides, we also discovered four overlapping nodes: moon, outside, delight and walk. Compared with the original network shown in Figure 1, our results can correctly cluster all nodes, verifying the effectiveness of our method.

### Performance on PPI networks

#### P-value

According to the SGD database, the P-value is an index to determine the statistical significance of the association of a particular GO term with a group of genes. It has been widely used in bioinformatics in recent years [4,26]. In general, its values are between 0 and 1. The closer the P-value is to zero, the more significant the particular GO term associated with the group of genes, i.e.:

(16) $$P-value=\frac{\left(\begin{array}{c} n\\ ol \end{array}\right)\left(\begin{array}{c} n-n_{2}\\ n_{1}-ol \end{array}\right)}{\left(\begin{array}{c} n\\ n_{1} \end{array}\right)}$$

where *n *represents the size of the entire network, *n*_1 _is a cluster obtained from the experiment, *n*_2 _the number of proteins annotated with a specific GO term and *ol *the number of proteins in *n*_1 _that can be annotated with the specific GO term.

In our experiments, P-values that higher than 0.01 were eliminated. We used the negative natural logarithms (-log P-value) to substitute for P-value.

### Cluster frequency

Cluster frequency is another index used in the SGD database which indicates the number of proteins in the experimental group annotated in a specific GO term. Although it is not as meaningful as P-value to represent the significance of a cluster to a specific GO term, its statistical value reflects the proportion of proteins that can reasonably be annotated, i.e.:

(17) $$cluster\;frequency=\frac{ol}{n_{2}}$$

### Discard rate

The discard rate represents the proportion of proteins not assigned to any module. In general, this rate reflects the filtering ability of the algorithm.

(18) $$discard\;rate=1-\frac{number\;of\;output\;data}{number\;of\;input\;data}$$

### Size distribution of PPI modules obtained by OMIM

**Figure 2:**
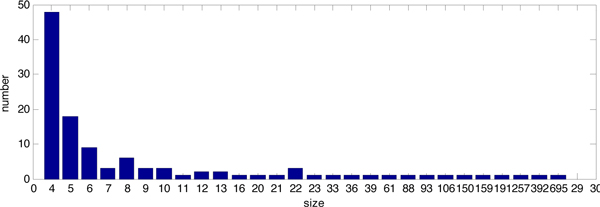
**Size distribution of PPI modules obtained by OMIM**. In Figure 2, the abscissa indicates the size of the modules, i.e, the number of proteins in each module. The ordinate shows the number of modules with the size corresponding to abscissa.

After setting the minimum module size to 4, we obtained 115 modules (Additional file 1) with a maximum value of *Q*=0.3616. Figure 2 is the size distribution of modules obtained by OMIM.

**Figure 3:**
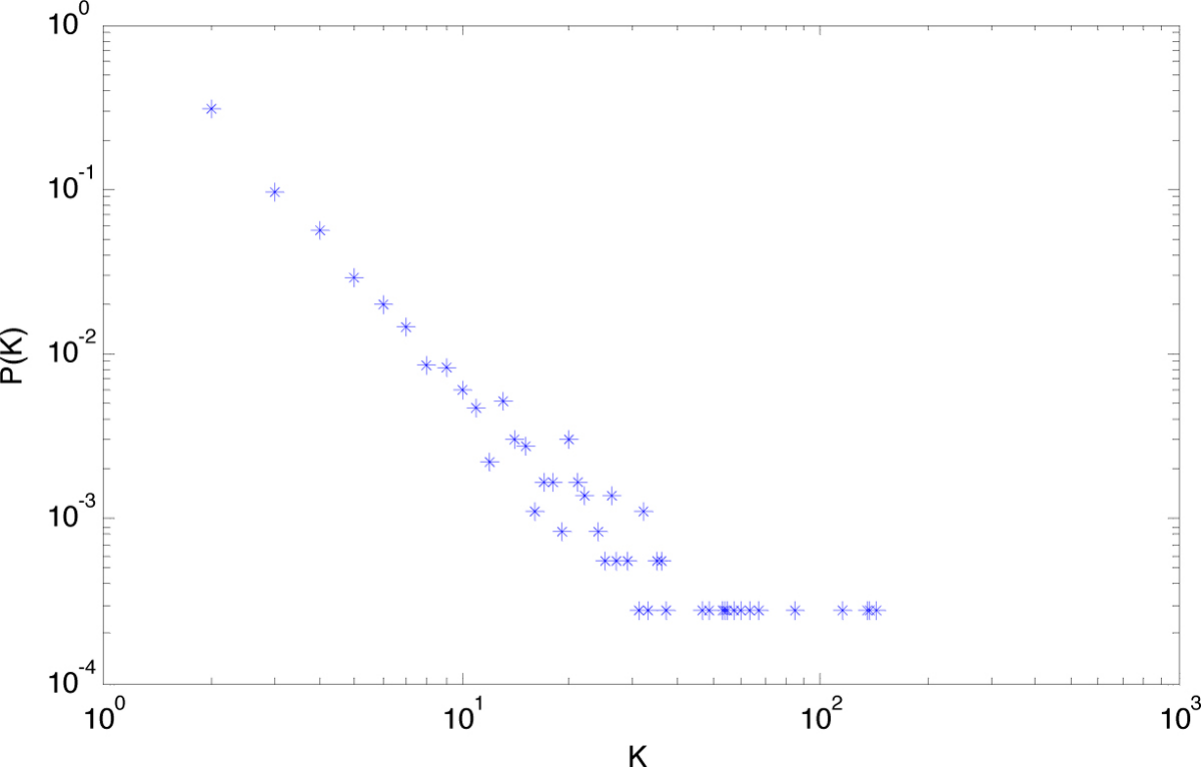
**Degree distribution of PPI dataset**. In Figure 3, K represents the degree of protein and the ordinate P(K) the fraction of proteins in the network with degree K.

Figure 2 shows that most modules are small, with very few modules that are extremely large. This coincides with the scale-free property of PPI networks, where most proteins interact with few partners, while a few proteins interact with many partners. The degree distribution of the PPI dataset in Figure 3 is able to explain the property.

From Figure 3 we can see that, like most scale-free networks, the degree of the distribution of the PPI dataset follows the power law relationship *P *(*K*)~*K*^-*r *^with *r≈*2.5.

### Enrichment analysis

Enrichment analysis is an important index for protein function annotation. We used the GO term finder to assign a main function that corresponding to the best P-value to each module. 10 modules were selected randomly to demonstrate the results of the enrichment analysis (Additional file 2).

Table 2 shows that most modules are able to be annotated to reliable functions on three Gene Ontology categories, i.e., molecular functions, biological process and cellular component. According to the P-values in Table 2, the most significant module is # 21, with -log P-values of 75.04, 44.07 and 83.63 respectively. However, there are also some modules which do not clearly belong to any GO term functions, such as module # 98. In addition, we can infer proteins with unknown functions according to their membership in a module. In module # 12, RRP4/RRP42/RRP43/SKI6 are with unknown molecular function. However, their neighbor, DIS3, has the following molecular functions: 3'-5'-exoribonuclease activity, tRNA binding and endoribonuclease activity. Consequently, we can infer that RRP4/RRP42/RRP43/SKI6 may be associated with one or more molecular functions of DIS3.

**Table 2:** Enrichment analysis of 10 randomly selected modules

Module	Protein	Main functions		
		
		BP(-log P-value)	MF(-log P-value)	CC(-log P-value)
3	CDC39/MOT2/NOT3/NOT5/	nuclear-transcribed mRNA poly(A) tail shortening (21.60)	ubiquitin-protein ligase activity (10.50)	CCR4-NOT core complex (24.36)
5	MSH2/MLH1/MSH3/MSH6/PMS1/	meiotic mismatch repair (31.64)	mismatched DNA binding (33.15)	mismatch repair complex (33.61)
11	SEN15/SEN2/SEN34/SEN54/	tRNA-type intron splice site recognition and cleavage (29.28)	endoribonuclease activity, producing 3'-phosphomonoesters (30.03)	tRNA-intron endonuclease complex (29.00)
12	DIS3/RRP4/RRP42/RRP43/SKI6/	nuclear polyadenylation-dependent mRNA catabolic process (27.68)	molecular function unknown (RRP4/RRP42/RRP43/SKI6)	cytoplasmic exosome (RNase complex (30.24)
21	CDC23/CDC16/APC9/APC4/APC2/APC11/APC1/APC5/CDC26/CDC27/DOC1/MND2/SWM1/	anaphase-promoting complex-dependent proteasomal ubiquitin-dependent protein catabolic process (75.04)	ubiquitin-protein ligase activity (44.07)	anaphase-promoting complex (83.63)
25	MRS11/TIM12/TIM22/TIM18/TIM54/TIM10/MRS5/TIM9/	protein import into mitochondrial inner membrane (38.73)	protein transporter activity (27.42)	mitochondrial inner membrane protein insertion complex (43.22)
26	TOM6/TOM5/TOM40/TOM20/TOM22/TOM7/TOM70/	protein targeting to mitochondrion (31.14)	protein channel activity (42.21)	mitochondrial outer membrane translocase complex (47.98)
98	YOL103w-b/PAN6/YOR142w-a/YER159c-a/YPR158w-a/	transposition, RNA-mediated (14.06)	RNA binding (6.37)	retrotransposon nucleocapsid (13.24)
103	TRS85/TRS33/TRS130/TRS20/GSG1/TRS65/TRS31/TRS23/TRS120/BET3/SED5/SLY1/BOS1/BET5/DSS4/YPT1/BET1/SEC34/YKT6/YPT6/SEC22/KRE11/	golgi vesicle transport (62.74)	rab guanyl-nucleotide exchange factor activity (35.95)	TRAPP complex (55.19)
115	rox3/sfl1/sin4/srb11/srb9/	positive regulation of transcription from RNA polymerase II promoter (9.93)	transcription factor binding transcription factor activity (15.39)	mediator complex (18.12)

Main functions: the GO term that obtained according to -log P-values of all modules for biological process (BP), molecular functions (MF) and cellular component (CC).

### Cluster frequency analysis

**Figure 4:**
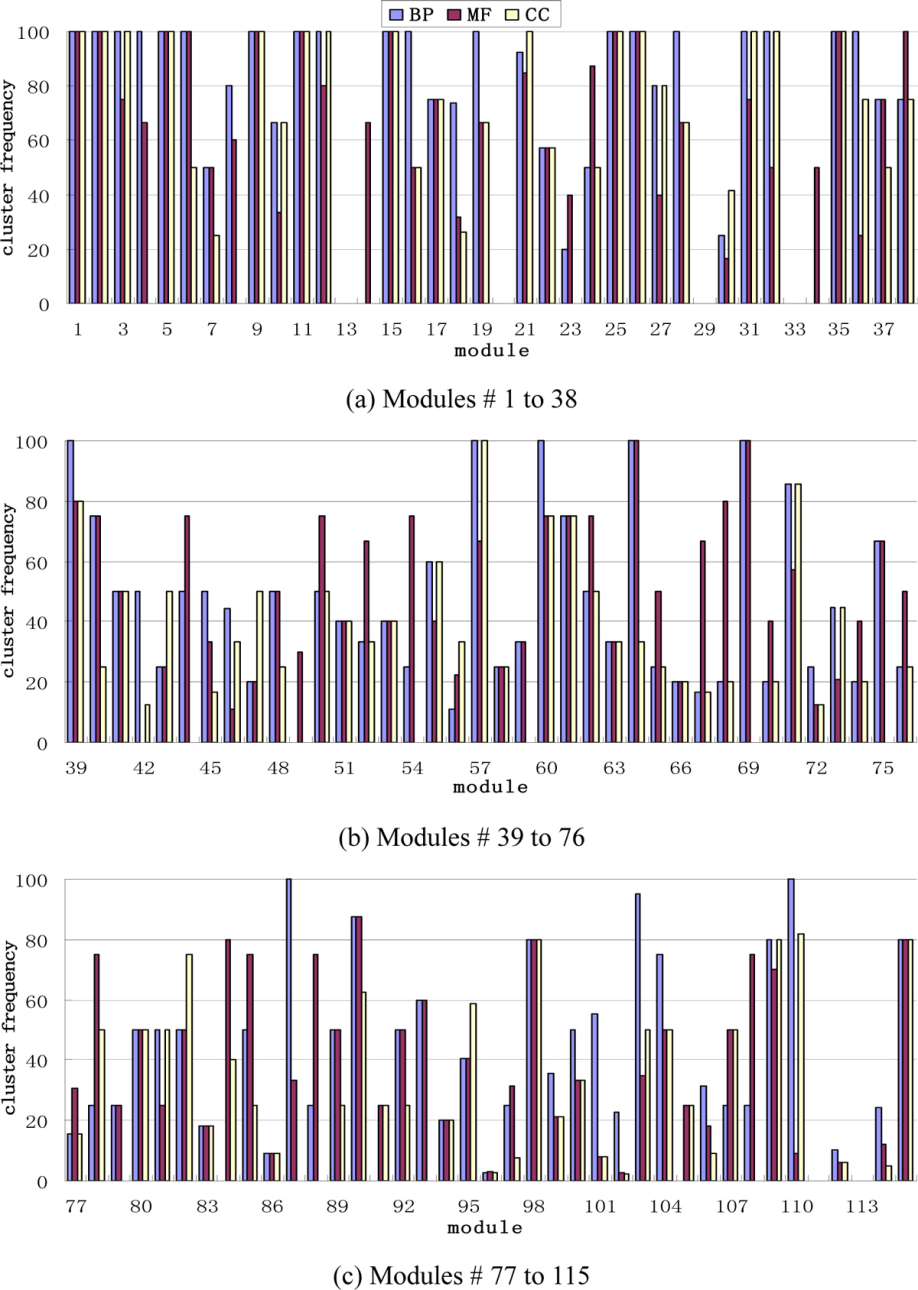
**Cluster frequency of 115 modules on category BP, MF and CC**. The abscissa indicates the module number and the ordinate the cluster frequency (%) in Figure 4. Cluster frequency on three main functions BP (biological process), MF (molecular functions) and CC (cellular component) were marked by different colors.

Cluster frequency analysis is another evaluation criterion for protein module construction, indicating the proportion of proteins in an experimental group annotated in a specific GO term (Additional file 2). Figure 4 is the cluster frequency of 115 modules obtained by OMIM. Figure 4 shows that most modules have a very high cluster frequency. In fact, 26 modules have a cluster frequency of 100% in the category of biological process. The result shows that most proteins in these modules have a common reliable function in OMIM.

### Comparison of OMIM with other algorithms on PPI dataset

In order to validate the OMIM on the PPI dataset, we compared it with the Newman, MCL and CPM algorithms. The results for the Saccharomyces cerevisiae PPI dataset are summarized in Table 3. The performance was largely measured by the discard rate and the enrichment analysis of Gene Ontology (molecular functions, biological process and cellular component).

**Table 3:** Comparison OMIM with other competing algorithms on PPI dataset

Algorithm	Module number	Module size	Discard rate (%)	GO(-log P-value)		
				
				BP	MF	CC
OMIM	115	25.81	44.26	7.27	7.69	7.44
Newman	115	24.18	44.26	7.18	7.39	7.22
MCL	319	7.40	52.68	7.17	6.72	7.16
CPM	66	10.96	85.51	8.39	7.60	8.53

Module number: the number of modules obtained by each algorithm. Module size: the average size of modules in each algorithm. GO: the average -log P-values of all modules for biological process (BP), molecular functions (MF) and cellular component (CC).

Table 3 shows that OMIM and Newman discard the least number of proteins (44.26%) for constructing modules compared with the other two methods. Moreover, OMIM is superior to Newman and MCL according to the enrichment analysis of Gene Ontology categories (BP, MF and CC). Although it has higher -log P-values on BP and CC than OMIM, CPM filtered too many proteins (about 85.51%) which may result in losing much useful information.

## Conclusions

The studies on an artificial and a PPI dataset verify the effectiveness of our method. In the experiment on the artificial dataset, the OMIM can find all modules correctly with an accuracy of 1.0000. All hubs that play key roles in the artificial networks are found precisely. In the experiment on the PPI dataset, we evaluated the performance of OMIM by enrichment analysis, cluster frequency analysis and in comparisons with other competing algorithms. All of the evaluation measures resulted in good performances. In addition, 30% of the hub proteins found by OMIM could directly be verified by the study of Han *et al*. [19]. However, since the degree distribution of the PPI dataset follows a power law, the discrepancy on modular sizes was quite large, which is not rational. In our future work, we will try to settle the problem of unbalanced clustering.

## Competing interests

The authors declare that they have no competing interests.

## Authors' contributions

XW and LL conceived the research and all authors designed it. LL carried out the calculations and all authors analyzed the results. The manuscript was drafted by LL and YC and written/revised by all authors. All authors approved the final version of the manuscript.

## Supplementary Material

Additional file 1 **A list of 115 potential functional modules.pdf**. This file contains all potential functional modules obtained by OMIM. For module #111 and 113, we did not list their members. The reason is that, their extremely large module sizes, 695 and 392, make them unreliable. Click here for file

Additional file 2 **Enrichment and cluster frequency analysis of 115 modules.pdf**. The best P-values and its corresponding cluster frequencies of 115 modules obtained by SGD Go term finder. The empty cells in this table denote 'No significant ontology term can be found for this module'. Click here for file
