# Supplementary material for: An overlapping module identification method in protein-protein interaction networks
Source: BMC Bioinformatics. 2012 May 8;13(Suppl 7):S4. doi: 10.1186/1471-2105-13-S7-S4 (PMC3348045; doi:10.1186/1471-2105-13-S7-S4)
Supplement: Additional file 1 — A list of 115 potential functional modules.pdf. This file contains all potential functional modules obtained by OMIM. For module #111 and 113, we did not list their members. The reason is that, their extremely large module sizes, 695 and 392, make them unreliable. [file 1471-2105-13-S7-S4-S1.pdf]

M01	ATP17	ATP4	ATP5	ATP6						
M02	CDC45	CDC46	CDC47	MCM3						
M03	CDC39	MOT2	NOT3	NOT5						
M04	PHO2	GRF10	PHO4	PHO80						
M05	MSH2	MLH1	MSH3	MSH6	PMS1					
M06	PMT1	PMT2	PMT3	PMT4						
M07	MLS1	ICL1	PYC1	PYC2						
M08	GAL3	GAL1	GAL80	GAL4	RPT6					
M09	CDC54	ORC2	ORC5	RRR1						
M010	SEC23	HIP1	SEC24	BFR2	SEC16	SEC31				
M011	SEN15	SEN2	SEN34	SEN54						
M012	DIS3	RRP4	RRP42	RRP43	SKI6					
M013	PEX15	RRD1	AGP3	SKI7						
M014	LCD1	DDC2	RNR1	SML1						
M015	SPC72	NUF1	SPC97	SPC98						
M016	NUP84	NEM1	NUP120	SPO7						
M017	GIN4	CDC3	CDC10	SHS1	7-Sep	CDC12	NFI1	CDC11	SPR28	
M018	GIP1	GAC1	PIG2	GLC7	BUD14	REF2	GAL83	PRP3	REG1	REG2
	SIP5	SNF1	SIP2	SIP1	SNF4	SSN3	SRB10	SSN8	CDC73	PAC10
M019	HHF2	HHT1	SPT6	HHF1	HHT2	STH1				
M020	CBC2	GCR3	MUD13	STO1						
M021	CDC23	CDC16	APC9	APC4	APC2	APC11	APC1	APC5	CDC26	CDC27
	DOC1	MND2	SWM1							
M022	AAC3	CCT4	CCT1	CCT2	CCT3	TCP1	SAC6			
M023	CIT3	ALG2	SSA4	DOS2	THI7					
M024	PCF11	RNA14	RNA15	PAB1	CAF20	CDC33	TIF4631	TIF4632		
M025	MRS11	TIM12	TIM22	TIM18	TIM54	TIM10	MRS5	TIM9		
M026	TOM6	TOM5	TOM40	TOM20	TOM22	TOM7	TOM70			
M027	CKA1	CKA2	CKB1	CKB2	TOP2					
M028	LST8	KOG1	TCO89	TOR1	BIT61	AVO3	AVO2	AVO1	TOR2	
M029	PRP40	MUD2	MSL5	PRP39	SMY2	SNF5	SPT15	BRF1	TFC3	TAF145
	KAP122	TOA1	TOA2	SRB2	RGR1	SIN4	GAL11	SNF2	SPT20	SWI1
	TAF25	LCP5	NGG1	SPT3	ADA2	ADR1	GCN5	HFI1	SPT7	SPT8
	TAF17	TAF60	TAF61	TAF90	TRA1	TAF40				
M030	AAT1	LEU2	SSY1	PTR3	SSY5	EFT1	EFT2	RET2	SEC26	BAP2
	TRK1	TRK2								
M031	TPS1	TPS2	TPS3	TSL1						
M032	Cdc15	Bub2	Bfa1	Tem1						
M033	BDF1	BDF2	MPS2	LPD1	OSM1	PUS2	FLX1	KRE28	MST1	BUD25
	AEP2	SVP26	GSC2	KAR2	IRE1	LHS1	NPL4	SEC63	CDC48	UFD2
	SNP1									
M034	GPI13	RPS20	MNE1	URA10						
M035	PEP3	PEP5	VPS16	VPS33						
M036	VPS20	SNF8	VPS25	VPS36						
M037	RAM1	CDC43	RAM2	VPS9						
M038	Pep12	SYN8	Snc1	Vti1						
M039	ALG5	OST1	STT3	SWP1	WBP1					

M040	PTC4	PTC3	PTC2	YDR071c						
M041	DBP6	DBP7	SPT14	YDR215c						
M042	ILV6	PUP3	PUP1	YDR428c						
M043	ACP1	SVS1	APT1	APT2	TIR1	RPL12A	YDR084c	YDR442w		
M044	DBP5	GFD1	YDL011c	YEL023c						
M045	SSP2	TRP1	LYS1	YEL064c	BIO3	YEL070w				
M046	DED81	HTM1	RPL30	SYGP-OR	DPB11	OYE2	POL2	SLD2	KRE32	DPB2
	YER083c									
M047	FAA3	RPS0A	SNU71	ALR1	YGL024w					
M048	RPM2	SEF1	VMA2	YGR117c						
M049	FIG1	ARGR2	GLK1	ARG2	SHE3	COX18	YDL193w	YDR516c	FRS2	YGR169c
M050	ADY1	HRR25	SPO13	PFS1	YIL007C	YIL007c				
M051	FRQ1	PIK1	IDH1	IDH2	YJL015c					
M052	PMN1	URH1	YIH1	YJL160c						
M053	CIS3	BUR2	SGV1	YGL042c	YJR030c					
M054	CHA4	YDL076c	SPO19	YKL047w						
M055	ARC1	MES1	OPI1	YGL245w	YKL098w					
M056	GTT1	TRM1	MEP1	EPT1	YEL017w	YJL097w	GTR1	GTR2	YKR007w	
M057	CDC13	HDF2	YKU70	HDF1	YKU80					
M058	PWP2	YGR154c	SEC62	YLR198c						
M059	YKR065c	YLR036c	YCR060w	YHR034c	FUN12	YLR241w				
M060	PST2	YBR052c	YCP4	YLR253w						
M061	RPP1B	RPP0	RPP2B	YLR287c						
M062	YGR004w	YDL089w	YIP2	YLR324w						
M063	BUD23	POP3	YFL044c	YFL043c	PAU4	YLR339c				
M064	(PUN1)	SHU1	YDR078c	YLR376c						
M065	RPL19B	YGR069w	YAT1	YLR381w						
M066	CTR3	POP1	SNM1	MLP2	MLP1	PML39	ISA1	RPS26a	YGL214w	YLR435w
M067	RPN5	YDR341c	YDR179c	YKR060w	IKI1	GPI16	YJR015w	TOT6	YLR327c	PHO89
	YLR137w	YML089c								
M068	PRM9	HOM2	YCR030c	YJR126c	YMR132c					
M069	SNA4	YDL114w	YLR040c	YDR348c	YMR295c					
M070	INO2	HCS1	INO4	PRM1	YMR317w					
M071	SNO3	SNO1	SNZ1	SNO2	SNZ2	SNZ3	YMR322c			
M072	GPA2	GPB1	GPB2	GPR1	RAD28	CBF5	YNL124w	LCB1	SLC1	YDR365c
	BUD3	BUD4	YIL104c	YKL224c	YLR030w	YNL326c				
M073	SMC4	IRR1	PRP21	NOG2	PRP11	PRP5	PRP9	RSE1	SPC25	NDC80
	NDC10	YDR295c	SPC19	SPC24	TID3	SPC42	NUF2	MCD1	SMC1	(TY4A)
	(TY4B)	SMC3	KAR5	SMC2	TRF4	YDL074c	YEL043w	YGR130c	YNR053c	MET28
	NNF1	KAR3	RAD50							
M074	LPP1	SLH1	YER049w	YIL169c	YNR074c					
M075	PRP12	REX2	YME1	YNT20						
M076	IPT1	YDR107c	THP2	YOL015w						
M077	YDR261w-	YCL020w	YDR261w-	BTT1	CAF130	EGD2	MFT1	YCL019w	YHR189w	HIT1
	RSA1	YJR003c	YOL030w							
M078	YLR143w	KRE29	YML023c	YOL128c						
M079	KTR7	RPL38	YBL089w	YOR082c						

M080	RNP1	YDR455c	YLR173w	YOR108w						
M081	PAN1	PAN2	YOL101c	YOR105w	ENT1	ENT2	ENT3	YOR111w		
M082	TSC10	YLR255c	AAD3	YOR114w						
M083	APL1	SBE2	SBE22	IML2	PPR1	STB6	YJR119c	MRPL49	PET112	YCR106w
	DPH2	YIL103w	CAT2	YNL046w	YNL018c	YOL075c	COX3	COX1	COX4	COX2
	OXA1	YOR121c								
M084	ISF1	MBR1	SAC1	SOK1	(ABP140)	YOR172w				
M085	RPC10	YIL025c	YOR006c	YOR352w						
M086	FAA4	NMT1	SPL2	YNL208w	YDR233c	RPS11A	DPM1	YOR285w	GCD10	GCD14
	YFL052w	YOR366w	ACC1	DMC1	PDC1	RDH54	RIS1	RPL11B	PDC5	YLR261c
	YOR390w	SNP1								
M087	SSK1	SSK2	PTP2	SLN1	YPD1	PBS2				
M088	SPI1	YCL063w	YJL043w	YPL005w						
M089	CDC19	PYK2	ISU2	YPL088w						
M090	PRT1	NIP1	RPG1	SUI1	TIF5	TIF35	TIF34	YPL105c		
M091	ATP20	CAJ1	HXT7	YPL205c						
M092	CIT2	MHP1	YNL285w	YPL245w						
M093	ALD5	GDA1	SSP120	YGL010w	YPL257w					
M094	APE2	NUT2	DIM1	PRY3	RCT1	YPL276w				
M095	IMP3	IMP4	MPP10	HSP104	YGR205w	YGR203w	YBL059w	BUD32	YCR099c	YIL059c
	NUP145	YGL060w	YHR036w	TAD2	TAD3	TIS11	PBP1	YHR121w	COQ2	CST29
	LAP3	YDL166c	HOM6	SEC13	THR4	YDR128w	SER2	TAF47	YKL177w	CDC36
	MSB2	YLR008c	YLR125w	YMR030w	LST7	PUT3	YDL110c	BUD21	YNL320w	CAN1
	UME1	YOR345c	PXA1	PXA2	LEU4	LOT5	ARO8	SMD1	PRP18	NAS6
	SLU7	YDL144c	RPB10	YDR527w	TOS5	YAL064w	CUP2	TPO1	YOR220w	SUN4
	YGR263c	YLR440c	FYV7	GAT1	SCL1	GIS4	NBP1	SAS3	YNL258c	RSC30
	YFL067w	DPS1	YOR282w	MSG5	NSR1	GDH1	ARP8	HKR1	VPS24	LST4
	IFM1	SKN1	PEX4	YMR211w	RRN10	RRN9	YIL141w	YJR087w	YBL010c	YKR022c
	YDL113c	YLR385c	YLR424w	DNL4	CDC50	LIF1	NEJ1	ENO2	YIL091c	YPR053c
	YJR082c	SAS10	YDL001w	CDC40	SNP1	SST2				
M096	YKL086w	AAH1	BTN2	YLR225c	PBN1	PRB1	YML031c-	LIA1	RMD9	YMR269W
	YOR309C	SDH3	SDH2	SDH1	TCM62	ETF-BETA	ETF-ALPH	YFH1	CBK1	YIR016w
	MOB2	YOL036w	ASG7	PET122	PET494	PET54	PET100	RPA12	RSM7	SDH4
	YAL045c	YAR047c	YBR099c	YBR116c	YBR219c	YBR220c	YDL129w	YDR196c	ADK1	HEM15
	POP7	RPP1	PPG1	YBR187w	PPT1	YER113c	YGL138c	PPH3	SDF1	PPH22
	TAP42	PFD1	YOR380w	HVG1	APS2	YMR119w	SPR6	YOL003c	SPO21	YDR136c
	ISC10	TRP5	PHO88	ICS2	FLO8	DER1	CIS1	RPB9	YAR064w	YBR089w
	YDL053c	YDL183c	YDL219w	YDR056c	YDR067c	YDR199w	YDR286c	YDR340w	YER181c	YJL182c
	YJR162c	YER053c	YLR326w	CCC1	SMB1	YNL187w	CPR8	YJL045w	YLR161w	QNS1
	YIL092w	YNR040w	YKL036c	YKL160w	YPK1	YIL023c	YOR154w	YPK2	EGT2	DOA4
	UBI4	YLL017w	YOR197w	HMS1	YIL108w	ADE5,7	MUM3	MCR1	MRD1	HMG2
	ABZ1	PIN3	PRE6	TCM1	YPL112c	YBR281c	YNL191w	YPL249c	FUN59	YGL051w
	PMA2	YKL174c	YPL136w	RGS2	YHL021c	YGR101w	YJL213w	MHT1	RIB4	YKR104w
	PLB3	WSC3	YBR014c	PHO86	BUD20	YBR101c	YKL171w	YLR194c	MSH4	MSH5
	YJR001w	YDR066c	SPB4	MSC6	COD4	YJR082c	YLR217w	YOL129w	DDR48	PBP2
	YPR096c	GAT1	PAT1	ATC1	KRE11	NTC20	HRB1	GNA1	YDR115w	
M097	SAP155	SAP185	SAP190	SIS2	SIT4	CDC55	GRR1	MET30	CDC6	CDC34

	RUB1	UBC12	UBA3	ULA1	CDC4	CDC28	CKS1	CLB1	CDC53	SIC1
	CLN1	BUD2	CLN2	CLN3	SSA1	YDJ1	SKP1	SGT1	YLR224w	SPH1
	FUS3	DIG2	DIG1	KSS1	STE12	STE11	STE5	STE50	STE7	HSP82
	CNS1	HSC82	STI1	STE18	FAR1	CTS1	GPA1	STE2	STE4	GCS1
	AKR1	YCK2	YCK1	YER079w	YDR425w	YGL198w	YBL049w	YIP1	YGL161c	YDR100w
	YPL095c	ESBP6	PWP1	GRE2	YGR111w	ALD6	BDP1	YGL250w	NET1	SIR2
	GPI2	ERG24	SAS10	YGR290w	YJL175w	YOL118c	YOR309c	YOR314w	YOR252w	YBR194w
	YPR152c	MPP10	CDC25	JSN1	MPT5	PBS2	CDC24	BUD6	BEM2	ELM1
	FYV13	CAK1	SKS1							
MO98	YOL103w-	PAN6	YOR142w-	YER159c	YPR158w-a					
MO99	CHK1	PHO91	SKI8	SKI3	SKI2	RPS28A	SRL2	YEL015W	PSU1	XRN1
	YEL015w	RRD2	YDL175C	YCR024C	SmD2	STE6	SMX1	RPS28b	RPS28a	NEO1
	MTR3	GCD11	DBF2	CDC15	DBF20	MOB1	DHH1	CCR4	POP2	RAS2
	RAS1	CDC25	SDC25	DCP2	PRP24	LSM8	HSH49	TOS8	DCP1	LSM1
	LSM4	PAT1	LSM6	LSM2	LSM3	RAD18	LSM5	LSM7	YFL066C	YJR138W
	YLR269c	YNR053C	YOR320C	YPR184W	SPR6	SSA1	PRP4	LTE1	KEM1	CIN8
	CDC45-1									
MO100	YER051w	YMR310c	YPS3	FUN16	FYV9	YNR046w	YIL151c	YPS4		
MO101	QCR8	ARK1	KRE9	YJR011C	YKL177W	FYV11	NFS1	RPB5	YLR243w	STM1
	YJR072c	YOR262w	YHR022c	YIL028w	YNR005c	YBR077c	YGR201c	ALD4	PPA1	SOD2
	MFA1	FYV4	YBR061c	YBR300c	YER028c	YGR219w	YGR235c	YGR277c	YGR182c	YPL066w
	RPB4	RPB7	YDR504c	YDR374c	YDR250c	YBL100c	YJL070c	YPS5	YDR383c	
MO102	PRS4	PRS2	PRPS1	OXR1	PRS5	YER163c	NCE103	CPA1	YMR134w	BAS1
	BAS2	SHM2	YGL242c	GDI1	AOS1	FMC1	PAN5	SNU114	PRP8	U5_SNRNa
	RAD53	STN1	ATC1	SSL2	PET127	AUS1	RNR2	DUN1	RNR4	MEC1
	ESR1	TEL1	MSL1	YDL001w	SEC14	YD8119.(	YDL133w	YHR197w	YNL165w	LEA1
	CHS1	YOR322C	YPL261C	YPR053C	CYP2	RPP1b	RPP2b	UGA2	UGA5	YCL010c
	CUP1-1	YKL023w	SRP40	YGR280c	SPS19	YMR102c	YNL218w	CDC91	SSA2	YIL040w
	YJR080c	YOR060c	FIR1	YJR110w	YMR093w	RPL32	YDR026c	FMN1	YDR398w	YPL216w
	YFR003c	YIL064w	YOR147w	YLR456w	YPR050c	RIB1	YNL105w	YPR172w	GRX3	RCS1
	YGL220w	YAP5	YGR168c	HOM3	PHM8	YIR014w	YKR079c	FPR1	SPP41	YMR087w
	CYS4	TFP1	PSR2	PSR1	WHI2	LRS4	SPO86	YDR061w	EFB1	TEF4
	TEF2	TEF1	YEF3	RNH70	SMM1	YLR334c	YKR090w	YKL146w	YJL055w	YIL060w
	YHR067w	YFR022w	NTG2	MSC7	IES1	BUD22	APN1	YOR264w	YOL034w	YOR324c
	CVT7	APG9	YLR065c	LCB3	PRM8	SLY41	ARO2	YOL107w	ACA1	YGR247w
	RTA1	ICY1	YOR059c	MUP3	ASP3B	TIF3	ISR1	CHS2	YCL022c	TRX2
	ULP1	YCR007c	YJL067w	NDD1	YDL203c	YGR058w	YGR136w	ERG6	PIS1	YCL023c
	DGA1	YGR228w	YOR097c	URA7	URA8	YDR133c	SEN1	REV7	MTD1	HXT17
	SMD3	YMR088c	YPR014c	CMD1	CMK1	CMP2	CNA1	CNA2	HPH1	SEC11
	SPC1	CMK2	SPC2	DAD2	YBR137w	ASK1	YKR083c	SGT2	YOL111c	ICT1
	YPS7	NBP1	RPB9	YDL100c	YNL091w	DST1	PCL7	FOB1	YJL215c	YNR068c
	YDR115w									
MO103	TRS85	TRS33	TRS130	TRS20	GSG1	TRS65	TRS31	TRS23	TRS120	BET3
	SED5	SLY1	BOS1	BET5	DSS4	YPT1	BET1	SEC34	YKT6	YPT6
	SEC22	KRE11								
MO104	MSO1	SEC1	PEP7	YPT52						
MO105	RPL5	YMR041c	YDL063c	YRA1						

M0106	PEX13	PEX17	PEX5	PEX14	PEX7	YGR010w	CAR2	YLR328w	SRA1	TPK2
	TPK3	APG7	AUT1	AUT7	YPR083w	YOL131w	YHR113w	PRI1	PEX3	MET4
	LAP4	LSB1	SAC2	VPS53	VPS54	SAS2	VMA6	SNF7	VPS4	YOR275c
	PRP45	PEP12	PRP46	APL6	APM3	VPS45	YLR345w	PRP4	BMH2	CHC1
	CLC1	YAP1801	YAP1802	YNL092w	APG16	RMT2	APG12	APG5	SAP18	SHE2
	HMO1	TAF19	TAF40	NUP157	NUP170	NUP82	YKL061w	YCR072c	SER3	FTR1
	YDR307w	DAM1	DAD1	DUO1	SPC34	APL2	APL4	APM2	DYS1	NUP192
	ASM4	NDC1	NUP53	NIC96	NUP188	IKS1	POM152	NSP1	LOS1	PUS1
	NUP49	YLR063w	SPO74	BUD5	FUN11	PEX19	NUP57	LTV1	BET1	YGR035c
	ATP14	RPP1A	SUA7	SUB1	YIL005w	YLR455w	BCY1	PKA3	TPK1	KAP104
	HRP1	NAB2	NUP100	GLE2	GLE1	NUP42	RIP1	YAP1	YBR216c	NUP116
	COR1	XDJ1	BUD8	YBR027c	SAP4	YOR062c	YNK1	YFR047c	TRR1	THI4
	SAE2	PSA1	PNC1	NUP1	IMD2	HEM2	FOL2	FBP1	CTA1	KAP95
	YIP3	KGD2	YLR108c	ECM15	SMT3	YCR087c-	JSN1	DID4	YEL068c	HSP10
	NUP2	SRP1	SOR1	YDL246c	YJR037w	RAP1	SIR3	SIR4	RIF1	HPC2
	RIF2	YNR071c	GTS1	THO1	YOR333c	SEC34	USO1	LYS14	NAP1	GRX5
	YIL105c	YMR068w	YNL047c	CSE2	ADE8	SRB7	RPB3	MED7	PGD1	MED2
	MED4	MED8	MED6	GIN11	RPL18B	BAT2	SPO12	IME1	RIM11	SRB4
	MEC3	SRN2	YLR051c	FAR3	FAR7	FAR8	FAR10	FAR9	MET28	SOH1
	MGE1	MDJ1	SSC1	PRO3	YMR124w	YFR008w	YJL218w	YHL018w	ILV1	HPA3
	HPA2	ECM31	CDD1	ASP1	AAD14	YOR284w	YJL199c	DCI1	ECI1	TEM1
	URK1	YJR056c	GCD7	ADY3	NIP29	YBR270c	YAP6	TFB1	YMR025w	PET123
	SEC35	APG13	YNL086w	APG17	COD1	YOR164c	YPL070w	YPL098c	GSP1	KAP123
	PSE1	CRM1	SRM1	RNA1	YRB2	TAF25	SMC1	YBL049w	LSM8	BET3
	YPT1	CVT19	PRE8	CBF1	HTA1	KNH1	PEA2	SLA1	BMH1	JNM1
	CSM3	RAD27	YKE2	GIM5	CDC7-1	CDC45-1	DDC1			
M0107	RVB1	RVB2	GLO4	YRO2						
M0108	BNA1	YML108w	KRE27	YSY6						
M0109	FIP1	CFT2	PTA1	CFT1	PAP1	UBA2	UFD1	PFS2	YSH1	YTH1
M0110	NOP1	NOP58	NOP5	SIK1	RRP1	RPF1	NSA1	NOP16	NOG1	NIP7
	MAK16	HAS1	EBP2	DRS1	BRX1	ERB1	NOP7	CIC1	NUG1	SPB1
	SSF1	TIF6	YTM1							
M0111										
M0112	OST5	ALG6	YDL096C	DFG5	RPO41	OST3	OST4	DIE2	MDM12	YGR064W
	DIA2	ALG8	YLR358C	PRM3	KRE6	POM34	PUS5	MRS7	YER064c	YJL075c
	ACE2	YNL157w	SUC2	YDR152w	FET4	YGR173w	YDL054c	YER140w	YKL056c	CYT2
	YLR285w	SER33	YLR053c	MET14	YNL311c	ERG3	FUR1	YHL049c	YOL099c	NOP13
	YHR180w	SCW10	YDL121c	YAH1	TTR1	MDH2	INO1	CYP5	CRH1	YDL100c
	YDR431w	YKR100c	YML101c	YOL159c	BIR1	YDR415c	YOR305w	MAM33	MRPS9	TIM17
	CYB2	TIM23	MAS6	TIM44	ERC1	CTH1	YDL012c	YHR140w	YIL172c	YJL065c
	YJL064w	YNR029c	IMD3	GDH2	PGI1	GCR2	TAH18	GDS1	INO80	YHL046c
	SPP2	DBP8	YKL075c	YNL091w	YNL164c	UBC4	UBC1	UBC5	QRI8	UBC8
	MIR1	UBC6	YPL229w	ERV2	TPO3	DFG10	YBR285w	ISY1	YGR003w	YLR297w
	CEF1	SYF1	SYF2	CDC40	PRP16	NTC20	GPX2	SYF3	AMI1	CLF1
	YBR190w	YDR013w	YBL006c	SRC1	YDR489w	YFR043c	YJL072c	YPL077c	YGR071c	CTM1
	YJL010c	YER093c	YJL058c	TBF1	YKL090w	YGR024c	YIL082w	YPL110c	RSA3	YMR187c
	YPR115w	PRP19	SNT309	YBL046w	ERV14	AAD6	YNL201c	PCH2	ZIP1	PRP18

	RPB9	MSL1	CYP2	YOL111c	FPS1	SNP1	YJU2	MRP4	GCN3	STD1
MO113										
MO114	RIM2	PRP31	YER078c	GYP8	YKL195w	NCA2	TOS1	YAP7	YBL095w	YNL217w
	CDC123	KRE22	YNL116w	UTR1	MRT4	YEL041w	SYGP-ORI	CST26	YHR115c	YOR215c
	RPL31B	YAE1	YDR492w	YOL035c	YOR315w	AKL1	PMT6	UBP5	ARG80	ARG81
	YML014w	AKR2	LCB4	GSP2	MOG1	YHR105w	YIF1	YOS1	FAA1	YBR064w
	HSP150	YBL044w	YDL023c	YNL321w	YBR239c	YMR144w	PIB1	YPL133c	FRE6	MTF2
	PSP1	CRC1	RSC1	PFK1	PFK2	UBP8	TFI1	RPS14B	TFI2	YDR115w
	YDR229w	YGL066w	YGR066c	PRE3	FYV2	ADE6	YLR386w	THI21	YHR207c	PKH1
	PKH2	YIR044c	YRF1-4	YNR004w	YPL157w	YPL004c	YTA6	YGR086c	MER1	MRP8
	SMP2	YMR210w	RPS7B	ZRC1	RPB9	YOR264w	KIC1	MGA1		
MO115	rox3	sfl1	sin4	srb11	srb9					
