# Supplementary material for: An overlapping module identification method in protein-protein interaction networks
Source: BMC Bioinformatics. 2012 May 8;13(Suppl 7):S4. doi: 10.1186/1471-2105-13-S7-S4 (PMC3348045; doi:10.1186/1471-2105-13-S7-S4)
Supplement: Additional file 2 — Enrichment and cluster frequency analysis of 115 modules.pdf. The best P-values and its corresponding cluster frequencies of 115 modules obtained by SGD Go term finder. The empty cells in this table denote 'No significant ontology term can be found for this module'. [file 1471-2105-13-S7-S4-S2.PDF]

BP:biological process (P-values)
MF:molecular functions (P-values)
CC:cellular component (P-values)
CF:cluster frequency

	BP	CF(%)	MF	CF(%)	CC	CF(%)
M01	1.08E-09	100	5.61E-09	100	1.62E-10	100
M02	1.58E-09	100	2.60E-09	100	4.09E-10	100
M03	4.16E-10	100	2.75E-05	75	2.63E-11	100
M04	3.56E-05	100	0.00204	66.7		
M05	1.81E-14	100	4.00E-15	100	2.53E-15	100
M06	8.19E-10	100	6.82E-13	100	6.07E-06	50
M07	6.16E-05	50	7.00E-07	50	unknown	25
M08	1.29E-09	80	2.27E-08	60		
M09	6.57E-07	100	2.66E-07	100	2.22E-07	100
M010	1.61E-09	66.7	0	33.3	2.36E-09	66.7
M011	1.91E-13	100	9.09E-14	100	2.54E-13	100
M012	9.53E-13	100	unknown	80	7.33E-14	100
M013						
M014			0	66.7		
M015	9.25E-10	100	2.71E-09	100	2.45E-10	100
M016	3.70E-07	100	0.00163	50	0.00013	50
M017	1.75E-11	75	1.56E-11	75	8.08E-14	75
M018	2.82E-06	73.7	9.92E-13	31.6	3.24E-12	26.3
M019	5.22E-11	100	0.00021	66.7	1.39E-09	66.7
M020						
M021	2.57E-33	92.3	7.27E-20	84.6	4.81E-37	100
M022	1.15E-05	57.1	7.16E-07	57.1	1.99E-09	57.1
M023	unknown	20	0.00E+00	40		
M024	5.77E-06	50	9.51E-06	87.5	9.19E-08	50
M025	1.51E-17	100	1.24E-12	100	1.70E-19	100
M026	3.00E-14	100	4.68E-19	100	1.45E-21	100
M027	8.11E-11	80	0.00014	40	1.18E-12	80
M028	4.12E-25	100	4.99E-05	66.7	1.96E-17	66.7
M029						
M030	2.29E-07	25	0.00107	16.7	0.00332	41.7
M031	6.36E-12	100	9.12E-10	75	8.18E-14	100
M032	3.40E-09	100	0.00073	50	1.88E-07	100
M033						
M034			0	50		
M035	4.64E-11	100	1.89E-08	100	3.95E-12	100
M036	7.45E-09	100	unknown	25	1.36E-09	75
M037	1.69E-08	75	9.12E-09	75	2.33E-06	50
M038	8.11E-05	75	2.90E-10	100	0.00033	75

M039	2.14E-10	100	2.86E-11	80	1.43E-10	80
M040	4.32E-05	75	1.42E-06	75	unknown	25
M041	0.0062	50	0.00123	50	0.00015	50
M042	0.00062	50			unknown	12.5
M043	unknown	25	0.00E+00	25	0.00797	50
M044	0.00857	50	unknown	75		
M045	0.00508	50	unknown	33.3	unknown	16.67
M046	5.24E-06	44.4	unknown	11.1	5.19E-07	33.3
M047	unknown	20	unknown	20	unknown	50
M048	unknown	50	0	50	unknown	25
M049			0	30		
M050	0.00272	50	unknown	75	0.00791	50
M051	0.00016	40	1.94E-06	40	9.73E-06	40
M052	unknown	33.3	unknown	66.7	unknown	33.3
M053	3.34E-05	40	unknown	40	0.00063	40
M054	unknown	25	unknown	75		
M055	4.40E-05	60	0.00097	40	2.11E-09	60
M056	unknown	11.1	0.0035	22.2	5.46E-07	33.3
M057	5.55E-05	100	4.49E-05	66.7	1.19E-06	100
M058	unknown	25	unknown	25	unknown	25
M059	unknown	33.3	0	33.3		
M060	unknown	100	unknown	75	1.13E-06	75
M061	0.00566	75	0.00025	75	0.0002	75
M062	0.00368	50	unknown	75	0.00035	50
M063	unknown	33.3	0	33.3	unknown	33.3
M064	6.84E-06	100	unknown	100	unknown	33.3
M065	unknown	25	unknown	50	unknown	25
M066	0.00047	20	0.00067	20	0.00392	20
M067	0.002	16.7	unknown	66.7	0.001	16.7
M068	unknown	20	unknown	80	unknown	20
M069	unknown	100	unknown	100		
M070	unknown	20	0.0003	40	unknown	20
M071	6.77E-18	85.7	0.0013	57.1	unknown	85.71
M072	1.24E-08	25	9.34E-05	12.5	unknown	12.5
M073	1.67E-13	44.8	3.88E-07	20.7	9.32E-16	44.8
M074	unknown	20	0	40	unknown	20
M075	0.00106	66.7	0	66.7		
M076	unknown	25	unknown	50	unknown	25
M077	0.00094	15.4	unknown	30.77	0.00024	15.4
M078	unknown	25	unknown	75	0.00013	50
M079	unknown	25	unknown	25		
M080	0	50	unknown	50	unknown	50
M081	1.71E-08	50	0.00021	25	3.88E-06	50
M082	unknown	50	0	50	unknown	75
M083	2.07E-06	18.2	4.86E-07	18.2	1.13E-06	18.2
M084			unknown	80	unknown	40

M085	unknown	50	unknown	75	unknown	25
M086	0.00822	9.1	0.00024	9.1	0.00043	9.1
M087	3.21E-14	100	2.10E-05	33.3		
M088	unknown	25	unknown	75		
M089	0.00017	50	1.16E-06	50	unknown	25
M090	1.46E-13	87.5	2.45E-16	87.5	1.34E-13	62.5
M091			unknown	25	unknown	25
M092	0	50	unknown	50	unknown	25
M093	unknown	60	unknown	60		
M094	unknown	20	unknown	20	unknown	20
M095	0.00129	40.4	unknown	40.38	3.31E-08	58.7
M096	0.00045	2.6	2.43E-06	3.2	0.00013	2.6
M097	2.93E-16	25	9.01E-11	31.5	2.16E-07	7.6
M098	7.78E-07	80	0.00171	80	1.78E-06	80
M099	1.00E-22	35.7	0.00095	21.4	2.01E-17	21.4
M0100	0.00249	50	0.00122	33.3	0.0011	33.3
M0101	unknown	55.26	0.00056	7.9	0.00117	7.9
M0102	unknown	22.78	0.00137	2.8	5.19E-05	2.2
M0103	5.69E-28	95	2.45E-16	35	1.07E-24	50
M0104	4.01E-07	75	0	50	0.00969	50
M0105			unknown	25	unknown	25
M0106	3.02E-23	31.5	7.01E-08	18.3	6.04E-18	9.2
M0107	unknown	25	0.00016	50	0.00016	50
M0108	unknown	25	unknown	75		
M0109	3.99E-19	80	5.23E-05	70	3.86E-20	80
M0110	1.54E-26	100	0.00113	9.1	2.76E-36	81.8
M0111						
M0112	1.89E-06	10.2	8.80E-12	6.1	4.21E-11	6.1
M0113						
M0114	unknown	24.1	0.00658	12	0.00021	4.8
M0115	4.86E-05	80	2.07E-07	80	1.35E-08	80
